# Single-Center Experience in Microsurgical Resection of Acoustic Neurinomas and the Benefit of Microscope-Based Augmented Reality

**DOI:** 10.3390/medicina60060932

**Published:** 2024-06-02

**Authors:** Mirza Pojskić, Miriam H. A. Bopp, Benjamin Saß, Christopher Nimsky

**Affiliations:** 1 Department of Neurosurgery, University of Marburg, 35037 Marburg, Germany; bauermi@med.uni-marburg.de (M.H.A.B.); sassb@med.uni-marburg.de (B.S.); nimsky@med.uni-marburg.de (C.N.); 2 Marburg Center for Mind, Brain and Behavior (MCMBB), 35032 Marburg, Germany

**Keywords:** microscope-based augmented reality, acoustic neurinoma, vestibular schwannoma, skull base surgery

## Abstract

*Background and Objectives*: Microsurgical resection with intraoperative neuromonitoring is the gold standard for acoustic neurinomas (ANs) which are classified as T3 or T4 tumors according to the Hannover Classification. Microscope-based augmented reality (AR) can be beneficial in cerebellopontine angle and lateral skull base surgery, since these are small areas packed with anatomical structures and the use of this technology enables automatic 3D building of a model without the need for a surgeon to mentally perform this task of transferring 2D images seen on the microscope into imaginary 3D images, which then reduces the possibility of error and provides better orientation in the operative field. *Materials and Methods*: All patients who underwent surgery for resection of ANs in our department were included in this study. Clinical outcomes in terms of postoperative neurological deficits and complications were evaluated, as well as neuroradiological outcomes for tumor remnants and recurrence. *Results*: A total of 43 consecutive patients (25 female, median age 60.5 ± 16 years) who underwent resection of ANs via retrosigmoid osteoclastic craniotomy with the use of intraoperative neuromonitoring (22 right-sided, 14 giant tumors, 10 cystic, 7 with hydrocephalus) by a single surgeon were included in this study, with a median follow up of 41.2 ± 32.2 months. A total of 18 patients underwent subtotal resection, 1 patient partial resection and 24 patients gross total resection. A total of 27 patients underwent resection in sitting position and the rest in semi-sitting position. Out of 37 patients who had no facial nerve deficit prior to surgery, 19 patients were intact following surgery, 7 patients had House Brackmann (HB) Grade II paresis, 3 patients HB III, 7 patients HB IV and 1 patient HB V. Wound healing deficit with cerebrospinal fluid (CSF) leak occurred in 8 patients (18.6%). Operative time was 317.3 ± 99 min. One patient which had recurrence and one further patient with partial resection underwent radiotherapy following surgery. A total of 16 patients (37.2%) underwent resection using fiducial-based navigation and microscope-based AR, all in sitting position. Segmented objects of interest in AR were the sigmoid and transverse sinus, tumor outline, cranial nerves (CN) VII, VIII and V, petrous vein, cochlea and semicircular canals and brain stem. Operative time and clinical outcome did not differ between the AR and the non-AR group. However, use of AR improved orientation in the operative field for craniotomy planning and microsurgical resection by identification of important neurovascular structures. *Conclusions*: The single-center experience of resection of ANs showed a high rate of gross total (GTR) and subtotal resection (STR) with low recurrence. Use of AR improves intraoperative orientation and facilitates craniotomy planning and AN resection through early improved identification of important anatomical relations to structures of the inner auditory canal, venous sinuses, petrous vein, brain stem and the course of cranial nerves.

## 1. Introduction

Acoustic neurinomas (ANs) or vestibular schwannomas (VSs) account for 4/5 of all tumors of the cerebellopontine angle and 9% of all intracranial tumors with typical symptoms of unilateral hearing loss, vertigo and tinnitus as well as hydrocephalus and brain stem symptoms in cases of larger tumors [1]. There are three possible surgical approaches for resection of these tumors: retrosigmoid, translabyrinthine and middle fossa approach [1]. Microsurgical resection with or without endoscopic assistance using intraoperative neuromonitoring and direct nerve stimulation for identification of the facial nerve (CN VII) [2] is the gold standard for tumors which are classified as T3 or T4 tumors according to the Hannover Classification, i.e., tumors which have contact with the brain stem [3,4,5]. Further options include observation (wait and scan) in cases of advanced age and smaller tumors without symptoms or comorbidities, as well as stereotactic radiosurgery, which provides local control but cannot cure the tumor [6]. Identification of CN VII as well as of relations of the tumor to the brain stem and neurovascular structures is critical for the facilitation of safe tumor resection. AR technology involves the superimposition of computer-generated images onto the user’s real-world visual field to modify or enhance the user’s visual experience [7]. The main principle is the segmentation of structures of interest using preoperative imaging, and the fusion of this dataset with the system for navigation, with projection of virtual images onto the binoculars of the microscope, the endoscope or onto a headset [8]. Microscope-based AR is used for improved orientation in the operative field in skull base surgery, using superimposed images of segmented structures of interest in a two-dimensional (2D) and three-dimensional (3D) manner. There is a hypothesis that AR is especially beneficial in cerebellopontine angle and lateral skull base surgery, since these are small areas packed with anatomical structures and the use of this technology enables automatic 3D building of a model without the need for a surgeon to mentally perform this task of transferring the 2D image seen in the microscope into an imaginary 3D image, which then reduces the possibility of error and provides better orientation in the operative field [9]. In this study, we demonstrated a single-center experience in the resection of ANs using conventional methods such as neuronavigation and intraoperative neuromonitoring and described our experience with the use of AR for facilitating resection of these lesions. To our knowledge, this is the largest study with the use of AR for resection of ANs. 

## 2. Materials and Methods

All patients who underwent surgery for resection of acoustic neurinomas in our department in the period January 2013–January 2024 were included in this study. Clinical outcomes in terms of postoperative neurological deficits and complications were evaluated, as well as neuroradiological outcomes for tumor remnants and recurrence. Facial nerve function was assessed using the House–Brackmann (HB) scale and it was measured at the point of last presentation. Preoperative and postoperative audiometry was performed in all cases to assess hearing function. 

The authors obtained ethics approval for prospective archiving of clinical and technical data from applying intraoperative imaging and navigation (study no. 99/18) from the local ethics committee at the University Hospital Marburg. Furthermore, upon our request in November 2022, the local ethics committee at the University Hospital Marburg considered an ethical approval unnecessary for this pseudonymized retrospective analysis (Az. 22/68).

Indications for surgery included acoustic neurinomas classified as T3 and larger according to the Samii (Hannover) classification [10]. Further indications included patients with T2 tumors who opted for surgery and rejected radiation therapy or the wait-and-see concept. Patients underwent surgery either in sitting position or in semi-sitting (Jannetta) position. Generally, patients with larger tumors underwent surgery in sitting position. Standard osteoclastic retrosigmoid craniotomy was performed. Invasive electrophysiological neuromonitoring with auditory evoked potentials (AEPs), motor evoked potentials (MEPs) and sensory evoked potentials (SEPs), as well as electromyography (EMG) of the facial nerve (CN VII) and trigeminal nerve (CN V), with intraoperative direct nerve stimulation (DNS), was always performed. All cases underwent resection using fiducial-based registration and neuronavigation. The first 27 cases were operated on using neuronavigation and intraoperative neuromonitoring, and the latter 16 cases underwent resection with additional microscope-based AR. The extent of resection was determined by postoperative MRI three months after surgery and was deemed as GTR (gross total resection), STR (subtotal resection) or PR (partial resection). 

### General Setup

**Figure 1:**
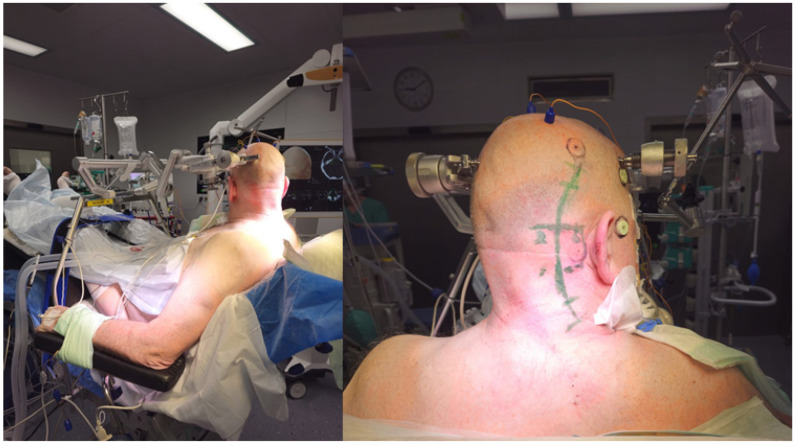
Operative setting. Patient is positioned in the sitting position, with the head tilted to the side of the lesion (Patient No. 43). Registration array is attached to the Mayfield clamp and is positioned to the right. Fiducial-based registration is performed prior to skin incision.

Patient was positioned in Jannetta (semi-sitting) or sitting position and head was fixed in a metal Mayfield head clamp (Figure 1).

**Figure 2:**
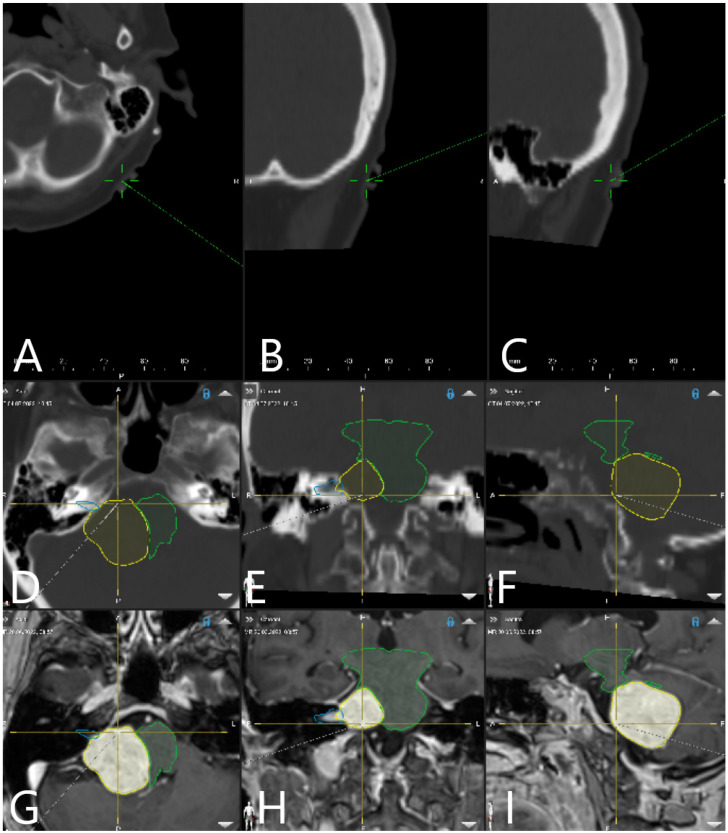
Patient No. 34. Preoperative MRI with segmented objects of interest was fused with preoperative navigation-CT with fiducials. Registration accuracy check on skin fiducial in (**A**) axial, (**B**) coronar and (**C**) sagittal view. Segmented objects of interest (tumor outline in yellow and brain stem in green) are presented in (**D**) axial, (**E**) coronar and (**F**) sagittal CT, as well as in (**G**) axial, (**H**) coronar and (**I**) sagittal T1-weighted post-contrast MRI.

In cases where preoperative MRI was performed with fiducials, fiducial-based registration was performed using this MRI dataset. In further cases, preoperative navigation-CT with fiducials was performed and fusion with MRI was performed (Figure 2).

**Figure 3:**
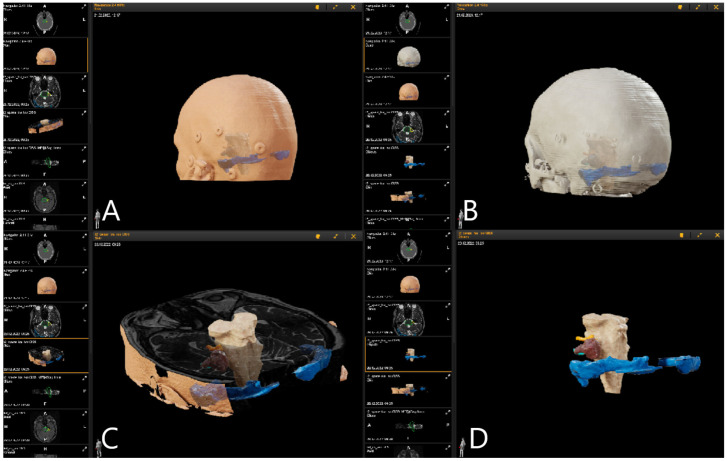
Preoperative approach and resection planning (Patient No. 39). Fusion of preoperative MRI and preoperative navigation-CT was performed. Objects of interest—tumor, trigeminal nerve, transverse and sigmoid sinus—were segmented manually and using autosegmentation with an anatomical mapping element. (**A**) Skin view for approach planning in sitting position with presentation of segmented objects and fiducials. (**B**) View of reconstruction of bony structures using the CT. (**C**) Reconstruction of segmented objects in T2-weighted CISS sequence. (**D**) Visualization of the tumor (brown), brain stem (white), trigeminal nerve (orange) and transverse and sigmoid sinus (blue) as a 3D object following segmentation in T2-weighted CISS sequence and T1-weighted post-contrast MRI.

T1-weighted post-contrast MRI modality was used for segmentation of the tumor, manually or using autosegmentation. Delineation of cranial nerves and semicircular canals of the inner ear was performed on T2 3D CISS sequence of the MRI, segmentation of transverse sinus and sigmoid sinus on T1-weighted MRI images, and for the brain stem usually on T2-weighted MRI, with different colors being assigned to each object, and with additional manual segmentation for correction of the automatic segmentation and for the segmentation of tumor and risk structures. Autosegmentation with an anatomical mapping element (Brainlab, Munich, Germany) can be additionally used for segmentation of anatomical structures (Figure 3).

**Figure 4:**
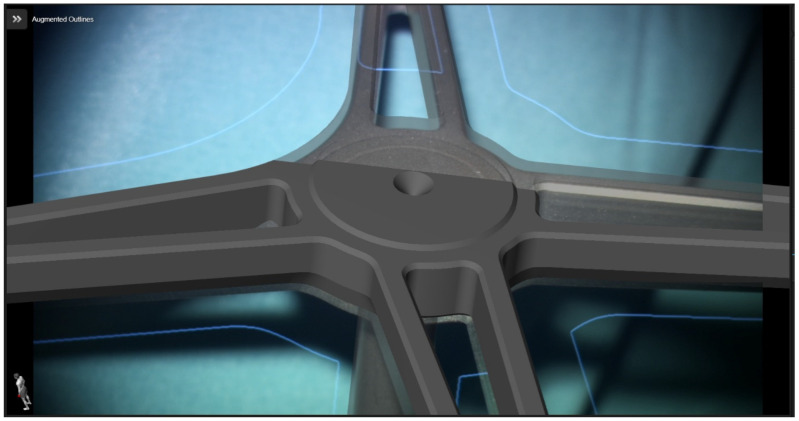
Calibration of the operative microscope (Patient No. 34). Outline of the registration array with AR-superimposed outline.

Following retrosigmoid craniotomy, calibration of the microscope was performed (Figure 4). For AR support, the HUDs of the operating microscopes (Pentero 900 (Zeiss, Oberkochen, Germany) or Kinevo 900 (Zeiss, Oberkochen, Germany)) were used. Tracking of the microscope was performed by a registration array attached to the microscope. Checking the calibration of the AR was performed by centering the microscope above the divot of the registration array, as well as additional markers. In this way, the optical outline and the AR visualization of the reference array could be adjusted, 3D objects could be visualized in semitransparent, solid or outlined mode using the AR display, and the microscope application allowed for visualization of these objects in different modes on the microscope video.

## 3. Results

Characteristics of the patients are summarized in Table 1. A total of 43 consecutive patients (25 female, median age 60.5 ± 16 years) who underwent resection of ANs via retrosigmoid osteoclastic craniotomy with the use of intraoperative neuromonitoring (22 right-sided, 14 giant tumors, 10 cystic, 7 with hydrocephalus) by a single surgeon were included in the study, with a median follow up of 41.2 ± 32.2 months. A total of 18 patients underwent subtotal resection, 1 patient partial resection and 24 patients gross total resection. A total of 27 patients underwent resection in sitting position and the rest in semi-sitting position. Out of 37 patients who had no facial nerve deficit prior to surgery, 19 patients were intact following surgery, 7 patients had House Brackmann (HB) Grade II paresis, 3 patients HB III, 7 patients HB IV and 1 patient HB V. In 6 patients who had CN VII deficits prior to surgery, 4 patients remained unchanged following surgery (1 patient with preoperative HB II, 2 with preoperative HB IV and 1 with preoperative HB V), and 2 patients worsened (both with HB II preoperatively and HB V postoperatively). Wound healing deficit with CSF leak occurred in 8 patients (18.6%). Operative time was 317.3 ± 99 min. One patient which had recurrence and one further patient with partial resection underwent radiotherapy following surgery (Patient Nos. 6 and 20). One further patient (Patient No. 33) with subtotal resection experienced recurrence 4 years following surgery, for which re-resection was recommended. A total of 16 patients (Patient Nos. 28–43, 37.2%) underwent resection using fiducial-based navigation and microscope-based AR (AR group), all in sitting position. The mean hospital stay was 10.7 ± 6 days. Table 2 summarizes the most important characteristics of the AR and the non-AR group.

Baseline characteristics of the two groups (operation with or without AR) did not differ: gender, body mass index (BMI), comorbidities, average tumor size and distribution across Koos and Hannover classifications. Patients who did not undergo surgery with AR were significantly older (65.11 vs. 51.21, *p* < 0.01) (Table 2). Operative time and clinical outcome, as well as the postoperative facial nerve function outcome, and the length of hospital stay, did not show differences between the two groups. Furthermore, there were no differences in operative time, clinical outcome and complication rate between patients who underwent surgery in sitting and in Janetta position (*p* > 0.01). One patient (Patient No. 30) had neurofibromatosis type 2 (NF-2) with bilateral acoustic neurinomas. Surgery was performed on the right side and the left-sided tumor underwent stereotactic radiotherapy.

All patients but three (Patient Nos. 22, 30 and 40) had an impaired hearing function prior to surgery. Patient Nos. 1, 4, 6, 8, 12, 14, 16, 19–21, 24, 25, 28, 29, 31, 33, 37, 39, 41 and 43 had a complete functional hearing loss prior to surgery. The rest of the patients had a hearing loss on the side of the tumor of 70–80%. Following surgery, all patients had a functional hearing loss.

The complication rate was higher in the non-AR group (*p* < 0.01). Overall complications included shunt-dependent hydrocephalus (Patient Nos. 1, 14, 25 and 27), CSF leak with wound healing deficit (Patient Nos. 2, 3, 17, 22, 27, 33, 39 and 42, in Patient Nos. 3 and 39 with rhinoliquorrho), pneumothorax (Patient No. 11) and postoperative chronic subdural hematoma (Patient No. 34). There were a total of seven patients with hydrocephalus. Patient Nos. 1, 14, 25 and 27 developed a shunt-dependent hydrocephalus and received a VP shunt following resection of the tumor. Patient Nos. 4 and 15 presented with hydrocephalus, which resolved following resection of the tumor. Patient No. 41 received a VP shunt prior to AN surgery due to hydrocephalus in an external institution. 

The segmented objects of interest in AR were sigmoid and transverse sinus, tumor outline, cranial nerves VII, VIII and V, petrous vein, cochlea and semicircular canals and brain stem, presented in the AR in 2D- and 3D-fashion. Use of AR facilitated the approach planning, craniotomy, dural opening, in-depth understanding of the tumor size and its relations to CN V, to vascular structures and to internal acoustic meatus. Craniotomy planning and dural opening was facilitated in all cases through identification of venous sinuses and their relations to the planned craniotomy and opening of the dura. Table 3 summarizes patients who underwent surgery with AR and the segmented objects. 

### Illustrative Cases

**Figure 5:**
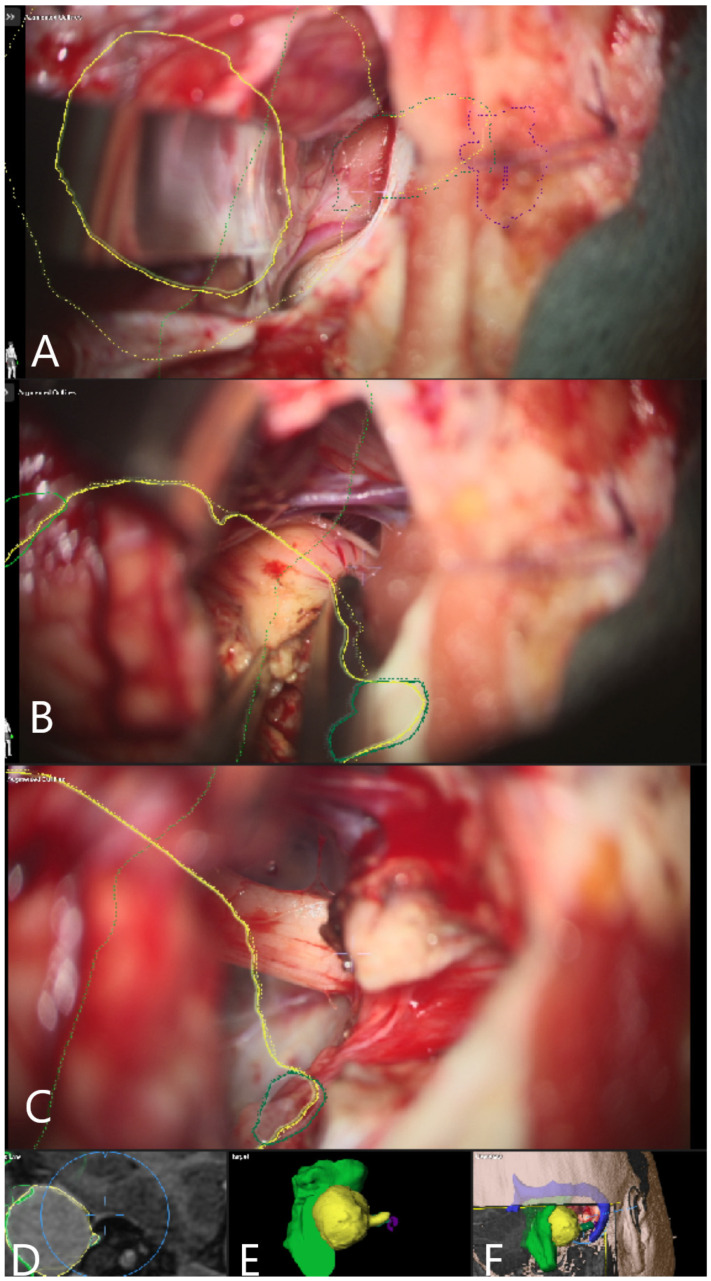
Patient No. 33. AR display on video screen with the 3D outline of tumor (yellow), brain stem (green) and venous sinus (blue). (**A**) Following dural opening, CSF release and initial exposure of the tumor. (**B**) During the course of capsule opening and debulking. (**C**) Following resection of the tumor with (**D**) corresponding probe’s eye view. (**E**) Target view (tumor and further objects outside of the focus plane are visualized) and (**F**) overview video plane in relation to the 3D objects.

**Figure 6:**
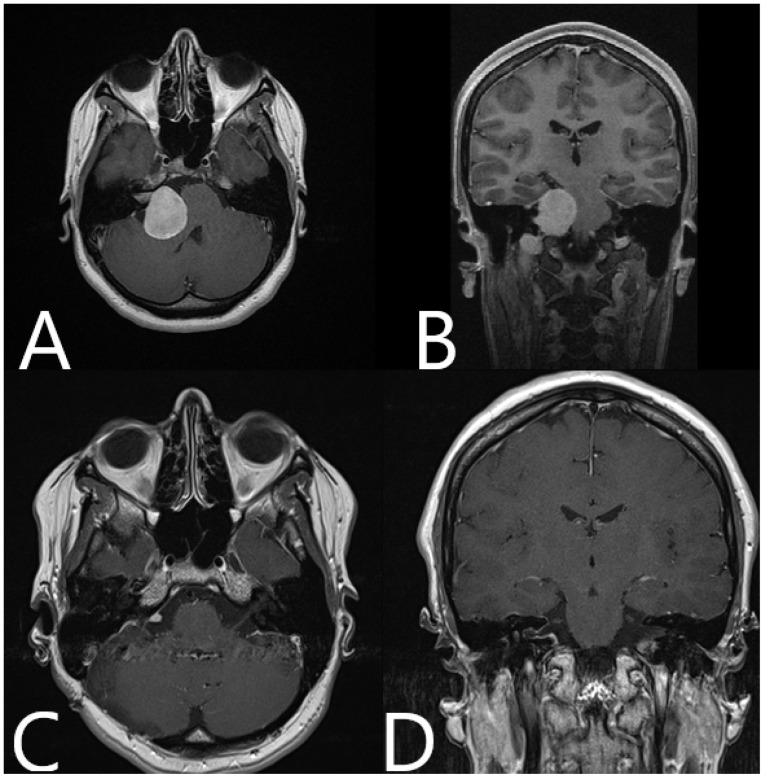
Patient No. 33. Preoperative (**A**) axial and (**B**) coronar T1-weighted post-contrast MRI with postoperative (**C**) axial and (**D**) coronar T1-weighted post-constrast MRI, depicting subtotal resection of the tumor and slight contrast enhancement along the course of the facial nerve.

Patient No. 33 was a 27-year-old patient with right acoustic neurinoma and a complete hearing loss on the right side and intact CN VII function. Subtotal resection of the tumor with AR support was performed, with small tumor remnants along the course of the facial nerve (Figure 5; Supplementary Materials—operative video). Postoperative CN VII function was HB Grade II. Follow-up MRI showed subtotal resection of the tumor with discrete contrast enhancement of the facial nerve (Figure 6).

**Figure 7:**
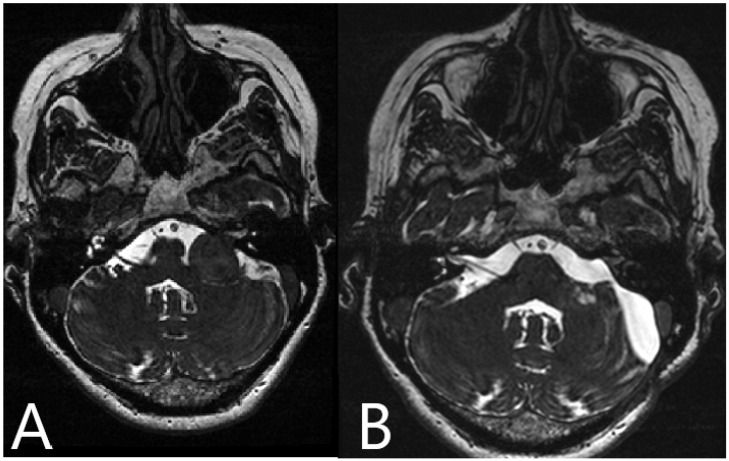
Patient No. 34. (**A**) Preoperative and (**B**) postoperative T2-weighted MRI of the head shows subtotal resection of the left sided AN.

Patient No. 34. (same patient as in Figure 2 and Figure 4) was a 49-year-old patient with T4a left-sided AN. She underwent subtotal resection of the AN with AR support (Figure 7). 

**Figure 8:**
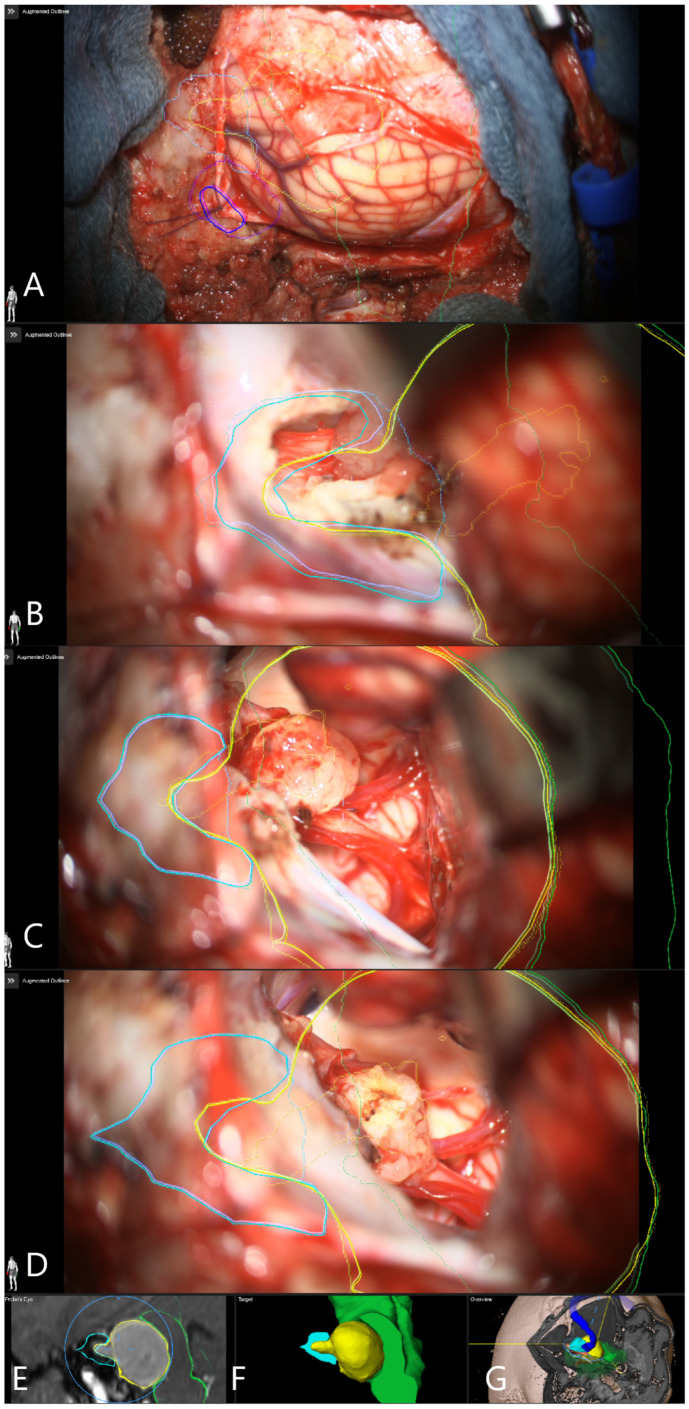
Patient No. 38. AR display on video screen with the 3D outline of tumor (yellow), brain stem (green), venous sinus (blue) and internal acoustic meatus (light blue) (**A**) following dural opening, (**B**) initial exposure of the tumor, (**C**) throughout the resection of the tumor with smaller remnant and (**D**) following resection of the tumor with small remnant on the facial nerve. Tumor remnant was left due to adherence to facial nerve and in order to preserve its integrity and function. (**E**) Corresponding probe’s eye view. (**F**) Target view (tumor and further objects outside of the focus plane are visualized) and (**G**) overview video plane in relation to the 3D objects.

**Figure 9:**
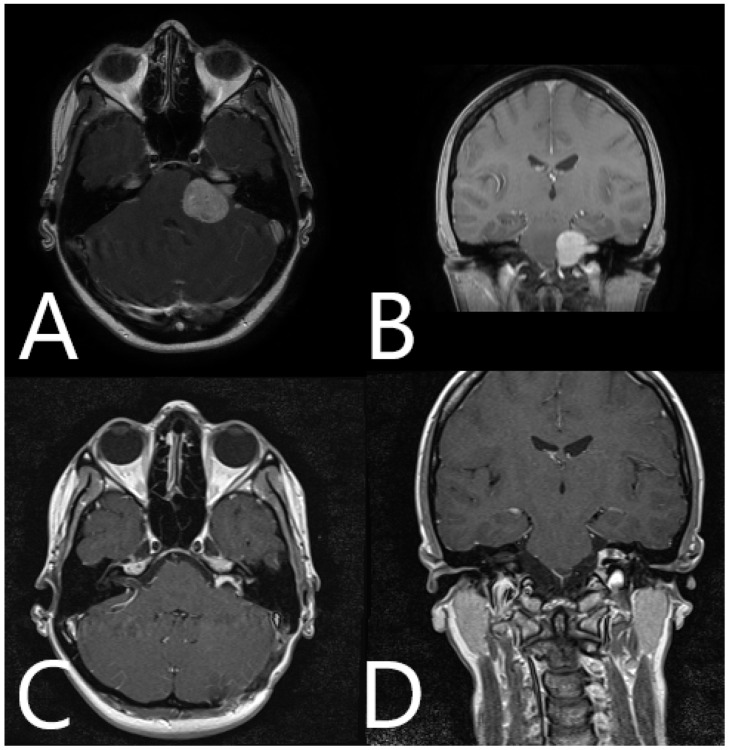
Patient No. 38. Preoperative (**A**) axial and (**B**) coronar T1-weighted post-contrast MRI with postoperative (**C**) axial and (**D**) coronar T1-weighted post-contrast MRI, depicting subtotal resection of the tumor and slight contrast enhancement along the course of the facial nerve.

Patient No. 38 was a 24-year-old patient with left sided T4B AN and functional hearing loss prior to surgery. Subtotal resection with AR support was performed. CN VII function was intact following surgery (Figure 8 and Figure 9).

**Figure 10:**
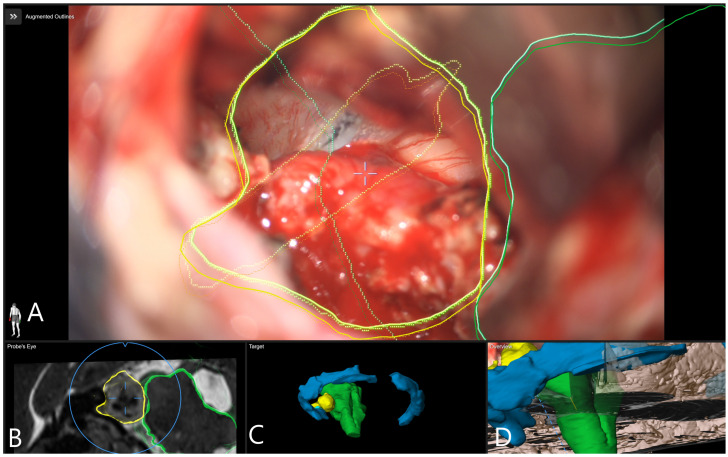
Patient No. 39. (**A**) AR display on video screen with the 3D outline of tumor (yellow), brain stem (green), venous sinus (blue) and internal acoustic meatus (light blue). (**B**) Corresponding probe’s eye view. (**C**) Target view (tumor and further objects outside of the focus plane are visualized) and (**D**) overview video plane in relation to the 3D objects.

**Figure 11:**
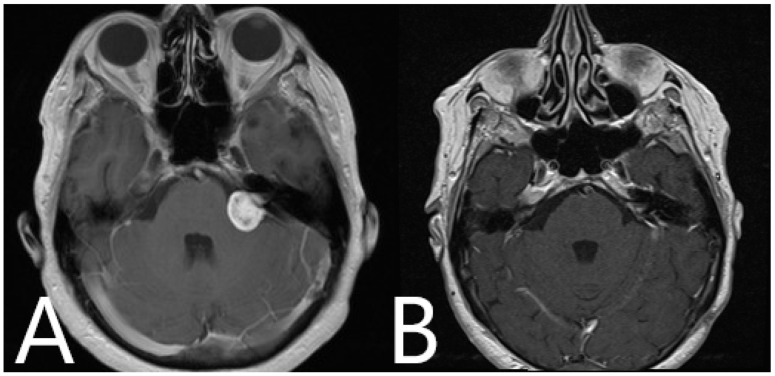
Patient No. 39. (**A**) Preoperative and (**B**) postoperative T1-weighted post-contrast MRI of the head shows subtotal resection of the tumor.

Patient No. 39 (same patient as in Figure 3) was a 72-year-old female patient who presented with complete hearing loss and headache. MRI revealed a T2 AN on the left side. She underwent STR of the tumor (Figure 10 and Figure 11).

**Figure 12:**
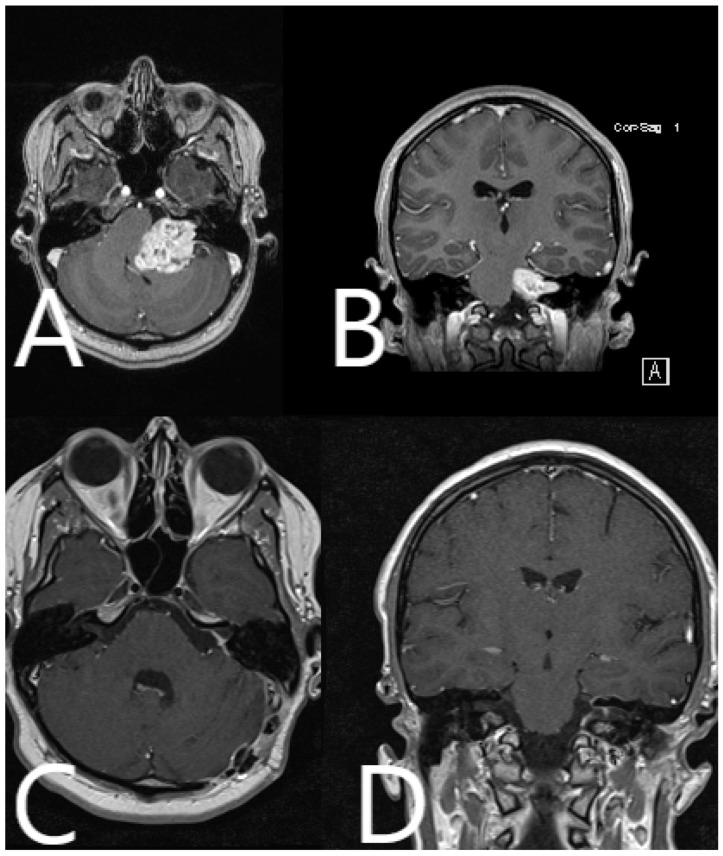
Patient No. 40. Preoperative (**A**) axial and (**B**) coronar T1-weighted post-contrast MRI with postoperative (**C**) axial and (**D**) coronar T1-weighted post-contrast MRI, depicting subtotal resection of the tumor and slight contrast enhancement along the course of the facial nerve.

Patient No. 40 was a 19-year-old female patient who presented with vertigo and headache. A giant T4B AN was subtotal resected. CN VII function following surgery was intact (Figure 12).

**Figure 13:**
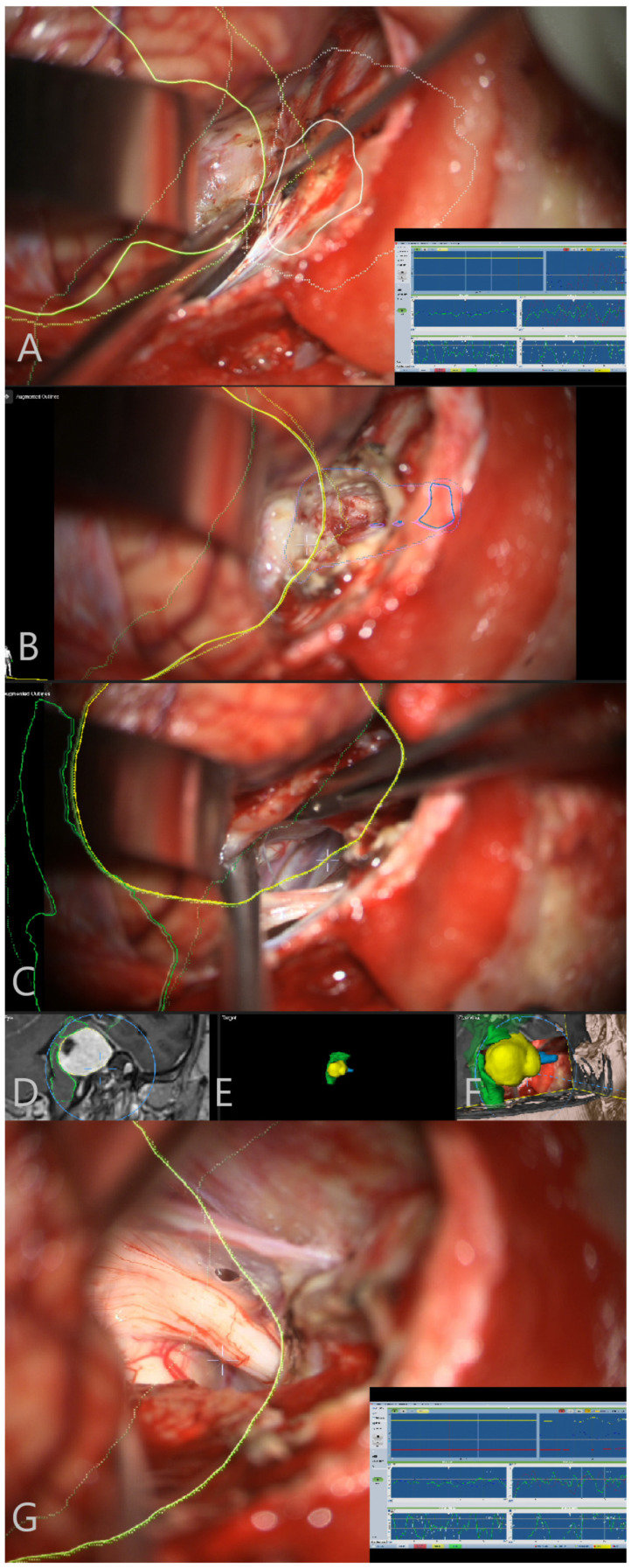
Patient No. 41. (**A**) AR display on video screen with the 3D outline of tumor (yellow), brain stem (green) and internal acoustic meatus (blue). Initial exposure of the tumor following CSF release and cerebellar retraction. CN VII direct nerve stimulation was performed and invasive electrophysiological neuromonitoring with EMG response was incorporated into microscope and display view, presented in the right lower corner. (**B**) AR display on video screen following drilling the roof of the internal acoustic meatus and (**C**) following GTR of the tumor with stimulation probe for direct nerve stimulation of CN VII at its brain stem origin. (**D**) Corresponding probe’s eye view. (**E**) Target view (tumor and further objects outside of the focus plane are visualized). (**F**) Overview video plane in relation to the 3D objects. (**G**) AR display on video screen depicting CN VII origin for near brain stem stimulation, with incorporated neuromonitoring display.

**Figure 14:**
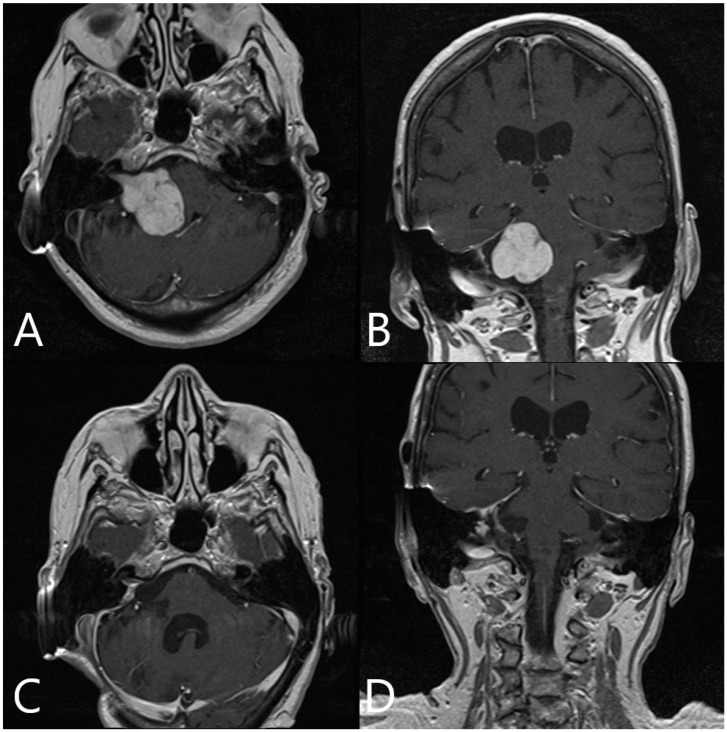
Patient No. 41. Preoperative (**A**) axial and (**B**) coronar T1-weighted post-contrast MRI with postoperative (**C**) axial and (**D**) coronar T1-weighted post-contrast MRI, depicting subtotal resection of the tumor and slight contrast enhancement along the course of the facial nerve.

Patient No. 41 was a 74-year-old patient with a giant T4b AN on the right side. She underwent gross total resection of the tumor. Prior to surgery, she had intact CN VII function. Following the surgery, she presented with HB III on the right side. At her last presentation, CN VII function was completely recovered (Figure 13 and Figure 14). 

**Figure 15:**
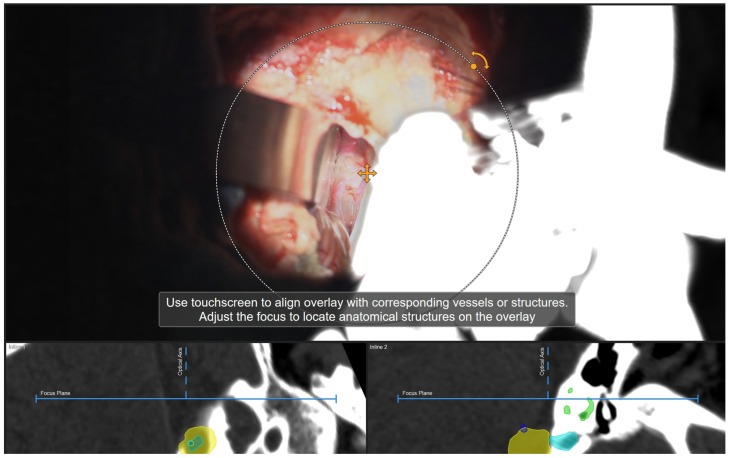
Patient No. 43. Microscope video display on the screen. Following retrosigmoid craniotomy, opening of the dura, CSF release, cerebellar retraction and initial exposure of the tumor, a navigation update was performed in the correlation to bony structures, i.e., roof of internal acoustic meatus. Segmented structures of interest included tumor outline (yellow), semicircular canals (green) and internal acoustic meatus (light blue). Navigation update enabled rotation of the microscope image with AR outlines parallel to bony structures in order to avoid any inaccuracies which might have emerged throughout craniotomy or CSF release.

**Figure 16:**
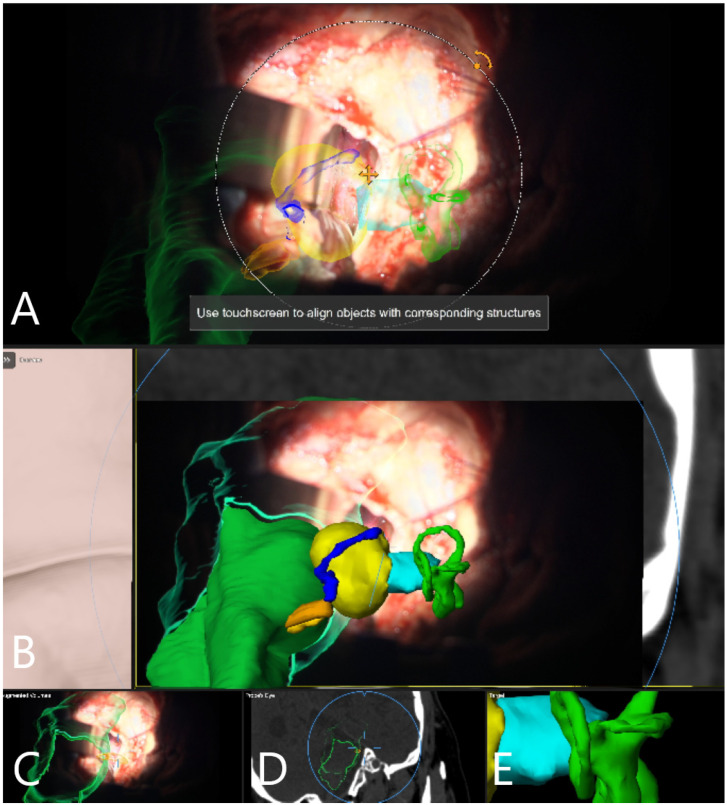
(**A**) AR display on video screen with the outline of tumor (yellow), petrous vein (blue), semicircular canals (green), CN VII/VIII complex origin at brain stem (orange) and internal acoustic meatus (light blue) following initial exposure of the tumor following CSF release and cerebellar retraction in 2D and (**B**) with segmented objects in full 3D volume. (**C**) Augmented outlines in 3D fashion. (**D**) Corresponding probe’s eye view. (**E**) Target view (tumor and further objects outside of the focus plane are visualized).

**Figure 17:**
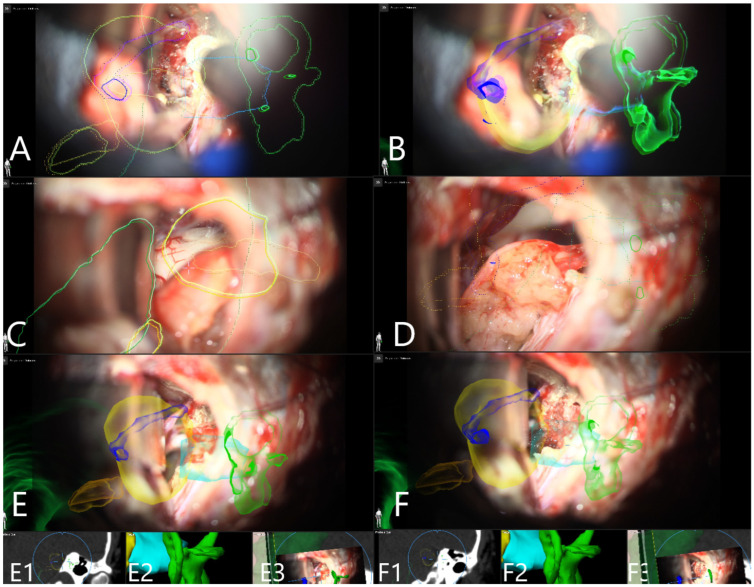
(**A**) AR display on video screen with the outline of tumor (yellow), petrous vein (blue), semicircular canals (green), CN VII/VIII complex origin at brain stem (orange) and internal acoustic meatus (light blue) during drilling of the roof of internal acoustic meatus in 2D and (**B**) 3D fashion. (**C**) Segmented structures are presented in 2D following exposure of CN VII in the internal acoustic meatus and (**D**) throughout the tumor resection with depiction of final steps of resection with small tumor remnant along the course of the facial nerve which was completely resected. (**E**) Segmented structures presented in 3D fashion at the end of the resection with (**E1**) probe’s eye, (**E2**) target view and (**E3**) overview video plane in relation to the 3D objects. (**F**) Last step of surgery with placement of muscle patch in the internal acoustic meatus, segmented structures in 3D fashion with (**F1**) probe eye’s view, (**F2**) target view and (**F3**) overview video plane in relation to the 3D objects.

Patient No. 43 was a 65-year-old male patient with a right-sided T2 AN who presented with complete loss of functional hearing. He underwent GTR of the tumor. CN VIII was HB III postoperatively. Use of AR enabled visualization of semicircular canals, and thus enabled safe drilling of the roof of the internal acoustic meatus (Figure 15, Figure 16 and Figure 17). 

## 4. Discussion

The main goal of surgery for ANs is maintenance and improvement of quality of life with preservation of CNVII and CNVIII function with proper oncological control [3]. In terms of resection extent as well as the use of radiotherapy, there is still controversy and no consensus to support any of several surgical strategies [3]. Intraoperative neuromonitoring needs to be routinely used to preserve neural function [3]. Intraoperative neuromonitoring includes obligatory measurement of the EMG of cranial nerves (CN V, VII and VIII) as well as auditory evoked potentials (AEPs), although pilot series on the use of a stimulation sucker for dynamic and continuous (space and time) CN VII mapping are reported [11]. 

All patients in our series were operated on using a retrosigmoid approach in sitting or semi-sitting position. Translabyrinthine and middle fossa approaches are alternatives, and the latter is suitable for hearing preservation surgery in the case of smaller tumors, whereas a retrosigmoid approach is associated with a higher risk of postoperative pain and CSF leak, yet shows the highest rate of facial nerve preservation for all tumor sizes [12]. The guidelines of the Congress of Neurological Surgeons (CNS) on resection of ANs state that there is insufficient evidence to support either the retrosigmoid or the translabyrinthine approach for complete resection of the tumor and CN VII preservation, when serviceable hearing is not present [2]. Only three patients in our series had intact hearing. A systematic review, which included 5064 patients, on complications in AN surgery revealed that the incidence of CSF leak was significantly greater after the retrosigmoid approach than after other approaches, with an incidence of 10.3% [12]. A recent study by Khan et al. which involved 415 patients reported a CSF leak incidence of 9.4% [6]. Wound healing deficit with CSF leak occurred in eight patients (18.6%) in this cohort. The increased incidence of CSF leak can be attributed to the fact that out of the eight patients with CSF leak, six had large tumors with cystic component, and two patients developed hydrocephalus, which manifested through CSF leak. There is a controversy surrounding patient positioning for AN surgery. However, a recent literature review which included 1640 patients deemed both positioning options—a lateral, supine position and a sitting/semi-sitting position—equally safe [13]. Furthermore, CN VII function had better long-term outcomes following surgery in sitting position, whereas the rate of venous embolism was higher, with equal perioperative mortality [13]. There were no cases of venous embolism in our cohort. 

An important parameter of clinical outcomes in patients with ANs is postoperative CN VII function. Six patients (13.95%) did not have intact CN VII deficit prior to surgery, which is a higher percentage than that described in the literature. CN VII palsy is an unusual symptom of ANs, which appears in less than 3% of patients, usually in patients of advanced age with hemorrhagic tumors [14]. Out of 37 patients who had no facial nerve deficit prior to surgery, 19 patients were intact following surgery, 7 patients had House Brackmann (HB) Grade II paresis, 3 patients HB III, 7 patients HB IV and 1 patient HB V. A total of 78.3% of patients with intact CN VII function (29/37) had an HB III or less following surgery, 70.2% HB < III and 51.3% were intact. This is compared to contemporary studies such as a study on the single-center experience of 72 patients who underwent surgery for ANs by Di Perna et al. [15], who identified 59.8% of patients as having a postoperative HB value < III, which reached 76.4% at the last follow-up evaluation. In all patients in our study, the integrity of CN VII was preserved. In 18 cases with subtotal resection, smaller tumor remnants were intentionally left on the CN VII in order to preserve the integrity of the nerve. This strategy, combined with postoperative radiotherapy (Gamma knife surgery) of the tumor remnant, was recommended as an optimal strategy for large ANs in a series of 47 patients, although long-term data in terms of the estimation of local control are missing [16]. Rinaldi et al., in their series of 66 patients with ANs, describe preservation of CN VII in 97% of patients, with a gross total and subtotal resection rate of 97% [17]. A large series of 200 patients who underwent resection using the retrosigmoid approach showed a CN VII preservation rate of 98.5% as well as excellent or good CN VII outcomes in 81% of patients [4]. Rates of CN VII preservation and good functional outcomes were lower in cases of giant ANs and were reported to be 75% in the series of Samii et al. [5]. A correlation of tumor size and CN VII outcome was found in other studies [17], whereas the extent of penetration in the internal auditory canal and surgical approach were not significantly correlated to CN VII outcome [17]. One further important outcome parameter which has emerged recently is hearing preservation. In our series, all patients but three had severely impaired hearing or complete hearing loss prior to surgery. Even in cases of smaller tumors with preserved hearing preoperatively, the use of hearing-preservation approaches has led to the loss of hearing during the follow-up period in 13% of cases and worsening in a further 55% [18]. Apart from studies from Europe, the largest North American study, which included 415 patients, reported poor CN VII outcomes in 14% of patients (HB III-VI), with tumor volume and preoperative CN VII function being considered as significant prognostic parameters, next to the CN VII stimulation threshold at the procedure and the consistency of the CN VII [6]. 

**Figure 18:**
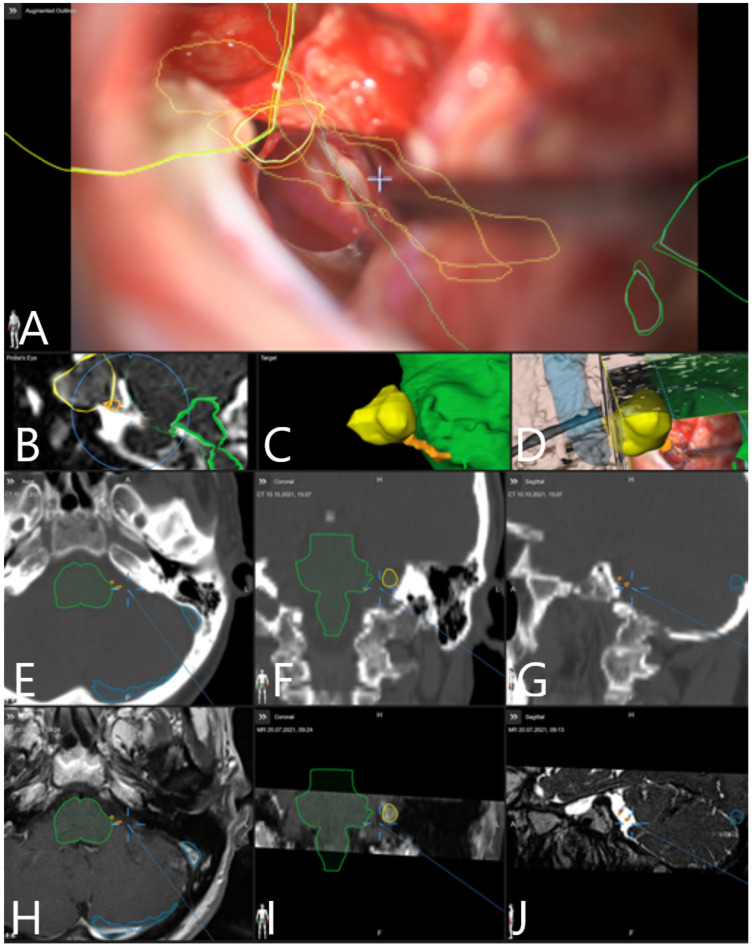
Patient No. 36, with left-sided AN and intact CN VII function. Patient underwent GTR of the tumor, and postoperative CN VII function was intact. (**A**) AR display on video screen with the outline of tumor (yellow), CN VII/VIII complex origin with course of CN VII at brain stem (orange) and brain stem (green). (**B**) Corresponding probe’s eye view. (**C**) Target view (tumor and further objects outside of the focus plane are visualized) and (**D**) overview video plane in relation to the 3D objects. (**E**) Navigation display with focus plane on the CN VII/VIII course with segmented objects (tumor outline in yellow, CN VII/VIII in orange, venous sinuses in blue) following craniotomy within CT mode in (**E**) axial, (**F**) coronar and (**G**) sagittal view as well as in (**H**) axial and (**I**) coronar T1-weighted post-contrast MRI and (**J**) sagittal view of T2-weighted MRI.

**Figure 19:**
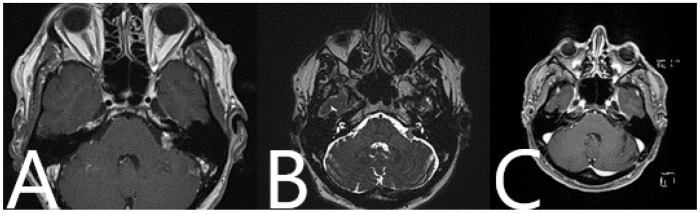
Patient No. 36. Preoperative (**A**) T1-weighted post-contrast and (**B**) T2-weighted MRI which shows a left-sided AN with contact to brain stem. (**C**) Postoperative T1-weighted post-contrast MRI shows complete resection of the tumor.

AR systems in cranial neurosurgery are designed to project 3D images of cerebral anatomy onto the real operative field, with superimposition of the images in a microscope (alternatively, a head-up display), without obstruction of the view, which assists by improving navigation and planning of the procedure, and leads consequently to a reduction in the operative time [19]. Surgical planning, localization of the brain structures, resection guidance and trajectory planning in neurovascular and neurooncological surgery have been previously described [19]. Craniotomy planning in skull base surgery is enabled through projections of the segmented structures of interest on the skin surface [20]. Retrosigmoid craniotomy planning was described in a cadaveric study [21], with projection of outlines of the dural sinuses on the skin and tailoring of the craniotomy. A recent literature review on the use of AR in skull base surgery identified nine clinical studies with 292 patients which thematized the use of this technology [22]. The primary data source was CT with optical tracking as the main modality (each 47.4%) and target registration error (TRE) which spanned from 0.55 to 10.62 mm [22]. Feasibility studies for AR use in lateral skull base surgery, which included the creation of a head-up display platform which allowed manual alignment of incorporated fiducial markers in real-time on a phantom model of a temporal bone using only CT rendering in the AR environment, were published in 2020 [23]; however, although AR visualization of CT could be transferred to surface landmarks of the patient, the target registration error was too high for proper use in surgery. However, Oemke et al. [24], using the same Brainlab software (Brainlab cranial software, 2019) as was used in this study, described safe and effective use of AR for the resection of tumors of the cerebellopontine angle, including ANs [8]. Schwam et al. [8] emphasized the value of AR in the preparation and approach planning for surgery of the cerebellopontine angle. Apart from segmentation of the tumor, the facial nerve, as well as the vestibulocochlear nerve, were labeled on preoperative imaging; however, due to tumor mass effect, there was a high possibility of an error in the labeling process due to overcrowding of these structures near the brain stem and in the internal auditory canal [8]. Incorrect segmentation and labeling of CN VII could be detected throughout the surgery using nerve stimulation [8]. Due to this described possibility of incorrect segmentation and the close proximity of CN VII and CN VIII in the cerebellopontine angle, we did not perform segmentation of a complete portion of these nerves but rather only their origin at the brain stem, and then intraoperatively detected CN VII using standard direct nerve stimulation. Identification of CN VII is possible in the internal auditory canal following its unroofing. Possible brain shift which occurs following cerebellar retraction and CSF release could be compensated and corrected on rigid bony structures of the lateral skull base, which can be used for navigation update [8]. Figure 18 and Figure 19 demonstrate AR-facilitated localization of CN VII and VIII origin at the brain stem. 

The use of AR in skull base surgery has been previously described. The use of AR for transsphenoidal resection of pituitary lesions, either in microscopic [25] or endoscopic [26,27,28] settings, has been implemented. In the setting of pituitary surgery, use of intraoperative CT and low-dose iCT protocols led to a significant reduction in effective dosage compared to a fluoroscopy-guided approach, and the use of AR has led to optimization of orientation in the surgical field, especially in cases of reoperations and anatomical variations [25]. As a prerequisite for optimal accuracy of the AR, automatic intraoperative imaging-based registration was recommended [25]. This is not possible, however, in cases of sitting and semi-sitting positioning of the patient, since an intraoperative CT scan cannot be performed. Intraoperative checks of the navigation accuracy, especially on the bony landmarks of the petrous bone and internal auditory canal, enable navigation update and thus control over AR accuracy. One further application of AR for skull base surgery is in reconstruction of the cranial vault as a single-step procedure following resection of complex skull base lesions [29]. A study on 3D models with defects involving the anterior skull base, with modeling of AR-assisted and manually shaped implants for the repair of the defect from PMMA (Palacos^®^R + G, Heraeus Medical GmbH, Wehrheim, Germany), has shown reduced deviation of the median volume from the profile of the surface of the original model in the case of an AR-assisted procedure compared to a “freehand” technique, with improved cosmetic results [29], which made this technique a viable and cost-effective alternative to patient-specific implants. 

Operative videos which depict the use of AR for resection of skull base meningiomas have been published, depicting its use in intraoperative orientation through estimation of tumor size, estimation of tumor remnant throughout the resection, as well as relations to neurovascular structures, especially in surgery in and around bony structures, such as for estimation of the extent of drilling in extradural clinoidectomy [22,30,31,32]. One important advantage of AR is that, even in cases of shift and slight registration inaccuracy, the size of a segmented object such as a tumor remains unchanged, which allows for estimation of the extent of resection. The localization of vessels, cranial nerves and brain stem and their relations to the tumor in this series provides in-depth perception and improves orientation. Furthermore, in cases of large tumors with encasement of the cerebral vessels, using AR during resection provides a good orientation and in-depth perception for the localization of the vessels inside the tumor, which reduces the risk of damaging these structures [33,34]. 

Besides its use in the operative room, AR has shown value as a training tool for the education of residents and young neurosurgeons in skull base surgery [35]. For educational purposes, Carlstrom et al. designed, from a skull base tumor registry, 3D models using high-resolution preoperative CT and MRI images, which were rendered for three possible surgical approaches following the uploading of 3D modeling data to an open-source database for virtual/augmented reality [36]. Surgical approaches such as mastoidectomy, anterior clinoidectomy and anterior petrosectomy were included in a training program using 3D-printed models with an AR system [37]. 

AR templates for surgery rehearsal for complex cases are possible future advances [30]. If an AR template which is tailored to the anatomy of an individual patient is used for surgery planning, this could mark a new era in microsurgical resection, where the surgeon would perform a surgery following “test surgery” on the model [38]. Specifically for surgery of the cerebellopontine angle, the use of diffusion tensor imaging (DTI) was described for the identification of cranial nerves (CN V, VII and VIII), with correlation to intraoperative findings of above 75% [39]. The so-called “probabilistic tractography” for tumors of the cerebellopontine angle was reported to have successfully identified all cranial nerves on the “healthy side” and 87% of displaced nerves on the side of the tumor, with adjustment of operative strategy in 71% of cases according to these findings [40]. Yet, prospective series with large numbers of patients are needed. In our opinion, the fact that DTI is a primary imaging of the functional tracts means its use in the proper identification of anatomical structures such as cranial nerves is limited; furthermore, the resolution of preoperative imaging is too low for accurate identification of the course of cranial nerves, especially inside the tumor. 

The limitations of this study include the low number of patients, although it was comparable to most contemporary studies due to the low-volume character of most university centers in Europe. Furthermore, this was a single-surgeon study, which reduces its reproducibility; however, prospective studies in educational and presurgical rehearsal settings for objective evaluation are needed. The group of patients which was operated on using AR was chronologically last and these patients had a shorter follow-up and underwent surgery with increased surgeon experience; however, our aim was to demonstrate the use of the technology as an adjunct to the existing standard practice and propose possible future advances. A further limitation was that the retrospective character of the study did not allow an attempt to express the improved comfort more objectively. Use of AR of en-block resection of spinal lesions has been previously described [41]. A recent prospective study by Roethe et al. attempted an objective assessment of the use of AR in the resection of cranial lesions using the Likert scale, and AR showed an improvement in the spatial understanding of information, visual accuracy of the overlay and transparency of the data source, and in visual comprehensibility [42].

## 5. Conclusions

This single-center experience of resection of ANs showed a high rate of GTR and STR with low recurrence. Clinical and radiological outcomes, as well as complication rates, showed results which are comparable to those in the current literature. Although there were no differences in clinical and radiological outcomes between patients who underwent surgery with and without the use of microscope-based AR, AR showed several benefits which improved orientation in the operative field: craniotomy planning through identification of transverse and sigmoid venous sinus, safe drilling of the petrous bone and roof of the internal acoustic meatus through early identification of semicircular canals and cochlea, as well as improved orientation in the operative field, especially in cases of larger tumors, through early improved identification of important anatomical relations to structures of the inner auditory canal, petrous vein, brain stem and arteries of the pontocerebellar angle, and relations to the course of cranial nerves. It is important to note that in this study there was no objective measurement of the use of AR and that increased surgeon comfort was a subjective matter. Further prospective studies are needed for the objective evaluation of AR use in this setting. In combination with the obligatory use of intraoperative neuromonitoring, this technique may contribute to safer resection of ANs. 

## Figures and Tables

**Table 1:** Characteristics of the cohort.

Patient Number	Age (yrs)	Gender	Tumor Volume (cm^3^)	Side, L = Left, R = Right	Position	AR	Hannover Classification	Koos Classification	Preoperative House Brackmann (HB) Grade of CN VII Function	Postoperative House Brackmann (HB) Grade of CN VII Function	Facial Nerve Outcome	Facial Nerve Outcome	Extent of Resection (PR = Partial, STR = Subtotal, GTR = Gross Total)
1	71	m	25.10	L	Jannetta	-	T4b	IV	HB II	HB II	I	Unchanged	PR
2	63	f	7.01	R	Jannetta	-	T4a	IV	HB I	HB II	I	Worsened	STR
3	60	m	6.25	R	Jannetta	-	T4a	IV	HB I	HB I	I	Unchanged	GTR
4	68	m	18.23	R	Sitting	-	T4b	IV	HB II	HB V	IV	Worsened	GTR
5	67	f	1.75	R	Jannetta	-	T3a	II	HB I	HB IV	IV	Worsened	GTR
6	79	f	7.37	R	Sitting	-	T4a	IV	HB IV	HB IV	IV	Unchanged	GTR
7	41	f	7.01	L	Sitting	-	T4a	IV	HB I	HB I	I	Unchanged	GTR
8	76	m	5.20	L	Sitting	-	T3a	III	HB I	HB I	I	Unchanged	STR
9	52	f	0.97	R	Sitting	-	T2	II	HB I	HB I	I	Unchanged	GTR
10	66	m	0.44	R	Sitting	-	T2	II	HB I	HB I	I	Unchanged	GTR
11	59	f	1.93	R	Jannetta	-	T3b	III	HB I	HB I	I	Unchanged	GTR
12	63	f	6.26	L	Sitting	-	T4b	IV	HB I	HB V	IV	Worsened	GTR
13	67	m	0.34	L	Jannetta	-	T2	II	HB I	HB I	I	Unchanged	STR
14	72	f	6.11	R	Jannetta	-	T4b	IV	HB I	HB IV	III	Worsened	STR
15	84	f	4.01	R	Jannetta	-	T4a	IV	HB I	HB II	II	Worsened	STR
16	72	m	32.80	L	Jannetta	-	T4b	IV	HB I	HB IV	III	Worsened	STR
17	60	f	0.59	R	Jannetta	-	T2	II	HB I	HB I	I	Unchanged	GTR
18	52	f	0.92	R	Sitting	-	T3a	II	HB I	HB I	I	Unchanged	GTR
19	76	f	4.37	L	Jannetta	-	T3b	III	HB I	HB III	III	Worsened	STR
20	50	f	2.14	L	Jannetta	-	T3b	III	HB I	HB I	I	Unchanged	STR
21	54	f	3.51	L	Jannetta	-	T3b	III	HB I	HB II	I	Unchanged	GTR
22	68	m	1.50	L	Jannetta	-	T2	II	HB I	HB I	I	Unchanged	GTR
23	39	f	1.39	L	Jannetta	-	T2	II	HB I	HB II	II	Worsened	STR
24	80	m	7.25	L	Sitting	-	T4a	IV	HB V	HB V	IV	Unchanged	GTR
25	60	m	28.10	R	Sitting	-	T4b	IV	HB II	HB V	IV	Worsened	GTR
26	84	m	6.74	R	Jannetta	-	T4b	IV	HB I	HB II	I	Worsened	STR
27	75	f	3.84	L	Sitting	-	T3b	III	HB I	HB I	I	Unchanged	STR
28	64	m	1.96	R	Sitting	AR	T3b	III	HB I	HB II	I	Worsened	STR
29	79	m	3.79	R	Sitting	AR	T3b	III	HB IV	HB IV	IV	Unchanged	STR
30	24	f	20.00	R	Sitting	AR	T4b	IV	HB I	HB IV	IV	Worsened	STR
31	61	f	1.63	L	Sitting	AR	T3a	II	HB I	HB I	I	Unchanged	GTR
32	55	f	1.14	L	Sitting	AR	T3a	II	HB I	HB I	I	Unchanged	GTR
33	27	f	13.50	R	Sitting	AR	T4b	IV	HB I	HB II	II	Worsened	STR
34	49	f	8.10	L	Sitting	AR	T4a	IV	HB I	HB IV	IV	Worsened	GTR
35	59	f	1.15	R	Sitting	AR	T3b	III	HB I	HB IV	IV	Worsened	GTR
36	49	m	1.78	L	Sitting	AR	T3b	III	HB I	HB I	I	Unchanged	GTR
37	60	m	0.76	L	Sitting	AR	T2	II	HB I	HB I	I	Unchanged	GTR
38	24	f	7.33	L	Sitting	AR	T4b	IV	HB I	HB I	I	Unchanged	STR
39	72	f	2.15	L	Sitting	AR	T2	II	HB I	HB IV	IV	Worsened	STR
40	19	f	14.60	L	Sitting	AR	T4b	IV	HB I	HB I	I	Unchanged	STR
41	75	f	15.00	R	Sitting	AR	T4b	IV	HB I	HB III	III	Worsened	GTR
42	72	m	16.10	R	Sitting	AR	T4b	IV	HB I	HB I	I	Unchanged	STR
43	65	m	3.30	R	Sitting	AR	T2	II	HB I	HBIII	III	Worsened	GTR

**Table 2:** Characteristics of non-AR and AR groups of patients with ANs.

Patient Characteristics	Non-AR Group	AR Group
Number of patients	27	16
Age	65.11 years	51.21 years
Gender	Male 12 Female 15	Male 6 Female 10
Hannover classification	T2 6 T3a 3 T3b 5 T4a 6 T4b 7	T2 3 T3a 2 T3b 4 T4a 1 T4b 6
Extent of resection	1 TR 15 GTR 11 STR	9 GTR 7 STR
Positioning	Sitting position 11 Semi-sitting position 16	Sitting position 16
Operative time	320 ± 86.4 min	310 ± 109.3 min
Hospital stay	11.7 ± 6.6 days	9 ± 4.2 days
Complication rate	9/27 patients Patient Nos. 2, 3, 17, 22, 25, 27—CSF leak with wound healing deficit Patient Nos. 1, 14, 25 and 27—shunt-dependent hydrocephalus Patient No. 11—pneumothorax	4/16 patients Patient Nos. 33, 42—CSF leak, wound healing deficit Patient No. 39—CSF leak, wound healing deficit, shunt dependent hydrocephalus Patient No. 34—postoperative contralateral supratentorial subdural hematoma
Preoperative CN VII function (HB grade)	22 HB I 3 HB II 1 HB IV 1 HB V	15 HB I 1 HB IV
Postoperative CN VII function (HB grade)	12 HB I 6 HB II 1 HB III 4 HB IV 4 HB V	7 HB I 2 HB II 2 HB III 5 HB IV
Postoperative CN VII function unchanged/worsened	16 unchanged 11 worsened	8 unchanged 8 worsened

**Table 3:** AR group of patients with segmented objects and applications throughout the procedure.

Patient Number	Segmented Structures in AR Advantages: Craniotomy Planning, Dural Opening, Relations of Tumor to CN V, Localization of Origin of CVI and CVIII at the Brain Stem, Relations of Tumor to Brain Stem, Localization of IV Ventricle, Segmentation of Tumor Cyst, Localization of Structures of the Middle Ear for Facilitation of Drilling on Inner Acoustic Meatus, Relations of Tumor to Arterial Vessels and to Petrosal Vein										
	Sigmoid Sinus	Transverse Sinus	Tumor Outline	CN VII and VII Origin at Brain Stem	CN V	Petrous Vein	Arterial Vessels (AICA, PICA, SCA)	Brain Stem	Middle Ear, Cochlea and Semicircular Canals	Pyramidal Tract	IV Ventricle
28	+	+	+	−	−	−	+	−	−	−	−
29	+	+	+	−	−	+	+	−	−	−	−
30	+	+	+	−	−	+	+	+	−	+	−
31	+	+	+	−	−	+	−	+	+	−	−
32	+	+	+	−	−	−	−	+	+	−	+
33	+	+	+	+	−	−	−	+	−	−	+
34	+	+	+	+	−	−	−	+	+	−	−
35	+	+	+	+	−	−	−	+	+	.	−
36	+	+	+	+	+	−	−	+	−	−	−
37	+	+	+	−	−	+	−	+	+	−	−
38	+	+	+	+	+	+	−	+	+	−	−
39	+	+	+	−	+	−	−	+	+	−	−
40	+	+	+	+	+	−	−	+	+	−	−
41	+	+	+	−	+	−	−	+	+	−	−
42	+	+	+	+	+	−	−	−	−	−	
43	+	+	+	+	+	+	−	+	+	−	−

## Data Availability

Portions of the data presented in this manuscript were presented as an oral presentation at the 18th World Congress of Neurosurgery in Cape Town, South Africa, under the title “Use of microscope-based augmented reality (AR) for microsurgical resection of acoustic neurinomas”, Skull Base II Session, Wednesday, 6th of December 2023. The title can be found online: https://pdf-program.abstractserver.com/?congress=wfns2023 (accessed on 1 April 2024). Portions of the data were also presented at the “4th Asian College of Neurological Surgeons—Bosnia and Herzegovina webinar”, 18 February 2023. Further data are available upon request from the corresponding author.

## Supplementary Materials

The following supporting information can be downloaded at: https://zenodo.org/records/10911722 (accessed on 28 May 2024). Operative video.
